# Supplementary material for: Yeast Sphingolipid-Enriched Domains and Membrane Compartments in the Absence of Mannosyldiinositolphosphorylceramide
Source: Biomolecules. 2020 Jun 6;10(6):871. doi: 10.3390/biom10060871 (PMC7356636; doi:10.3390/biom10060871)
Supplement: Supplementary file 1 [file biomolecules-10-00871-s001.pdf]

Supplementary Material

Yeast sphingolipid-enriched domains and membrane compartments in the absence of mannosyldiinositolphosphorylceramide

Andreia Bento-Oliveira[1], Filipa C. Santos[1], Joaquim Trigo Marquês[1], Pedro M. R. Paulo[2], Thomas Korte[3], Andreas Herrmann[3], H. Susana Marinho[1], Rodrigo F.M. de Almeida[1,*]

[1]*Centro de Química Estrutural, Faculdade de Ciências, Universidade de Lisboa, 1749-016 Lisbon, Portugal*

[2]*Centro de Química Estrutural, Instituto Superior Técnico, Universidade de Lisboa, 1049-001 Lisbon, Portugal*

[3]*Department of Biology, Molecular Biophysics, IRI Life Sciences, Humboldt-Universität zu Berlin, Berlin, Germany*

*Correspondence: rfalmeida@fc.ul.pt; Tel.:+351217500925

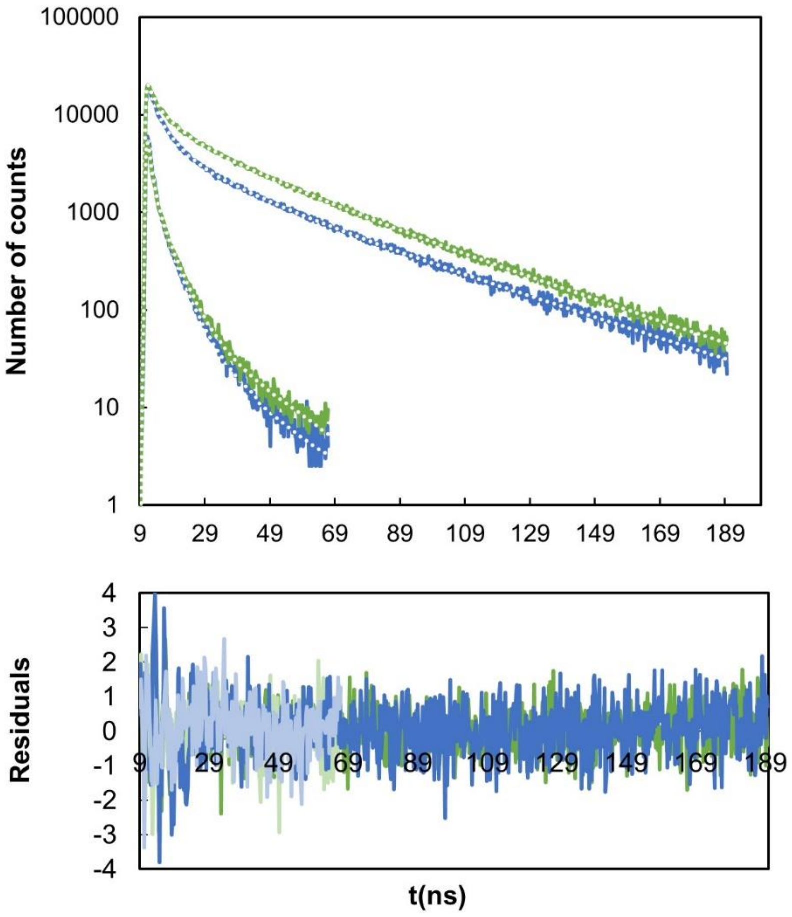

Figure S1: Fluorescence intensity decay of *t*-PnA in *wt* (blue) and *ipt1*Δ (green) cell suspensions at 24 ºC. Top panel: experimental decays in *t*-PnA labelled and unlabeled cell suspensions (faded blue and green, respectively) and best fitting function with a sum of exponentials (white dashed line) obtained with a global analysis method. Bottom panel: Residues. *t-PnA* fluorescence decays were obtained as described in the experimental procedures.

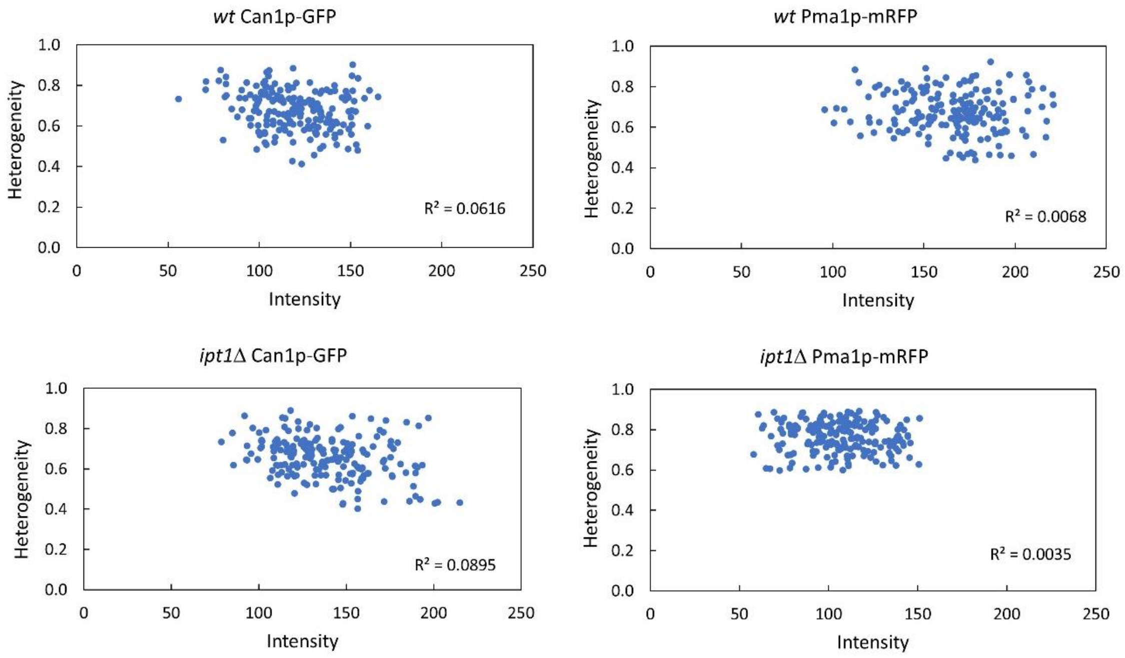

Figure S2: Relation between the average fluorescence intensity of Can1p or Pma1p along the plasma membrane and their distribution heterogeneity. Heterogeneity has no correlation with the intensity of the signal (in arbitrary units), which is not only confirmed by the dispersion of the results throughout the graph but also by the very low value of the linear determination coefficients. Each data point corresponds to one cell.

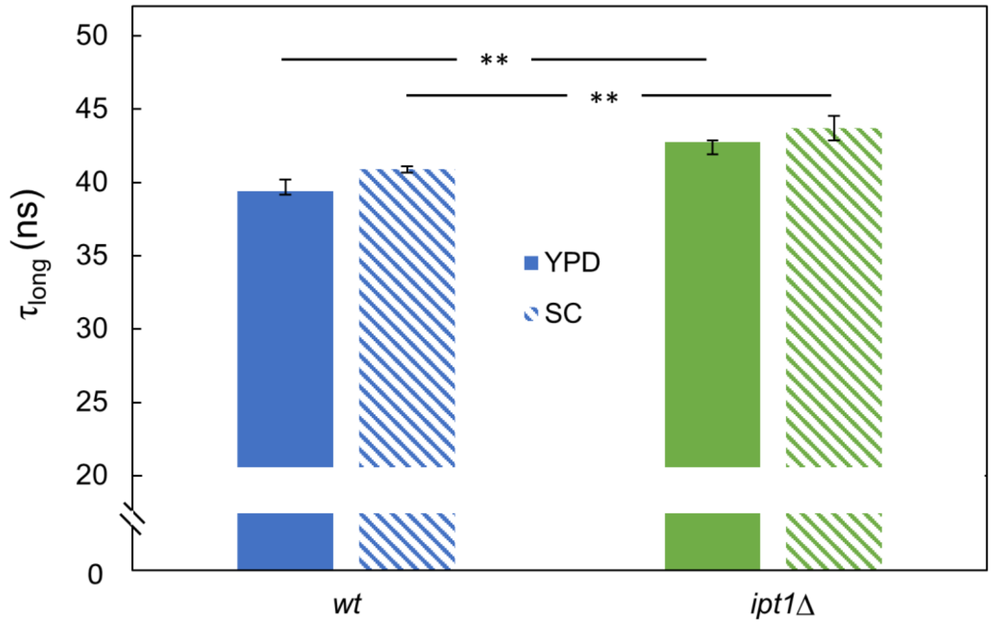

Figure S3: The lifetime of the long component of *t*-PnA is essentially identical in growth media SC and YPD (yeast extract, peptone, dextrose). The fluorescence intensity decay of the probe in *S. cerevisiae* cellular suspensions at 24 °C was obtained as described in the "experimental procedures" for living *wt* and *ipt1*Δ cells in mid-exponential phase grown in SC or YPD (1 % (w/v) yeast extract, 2 % (w/v) peptone, 2 % (w/v) glucose) media. For both strains, the long component lifetime is tendentially shorter for cells grown in YPD. Nonetheless, the long component lifetime is always statistically longer in *ipt1*Δ than in *wt* cells. The values are the mean ± S.D. of at least four biological replicates. **, $p \leq 0.01$.

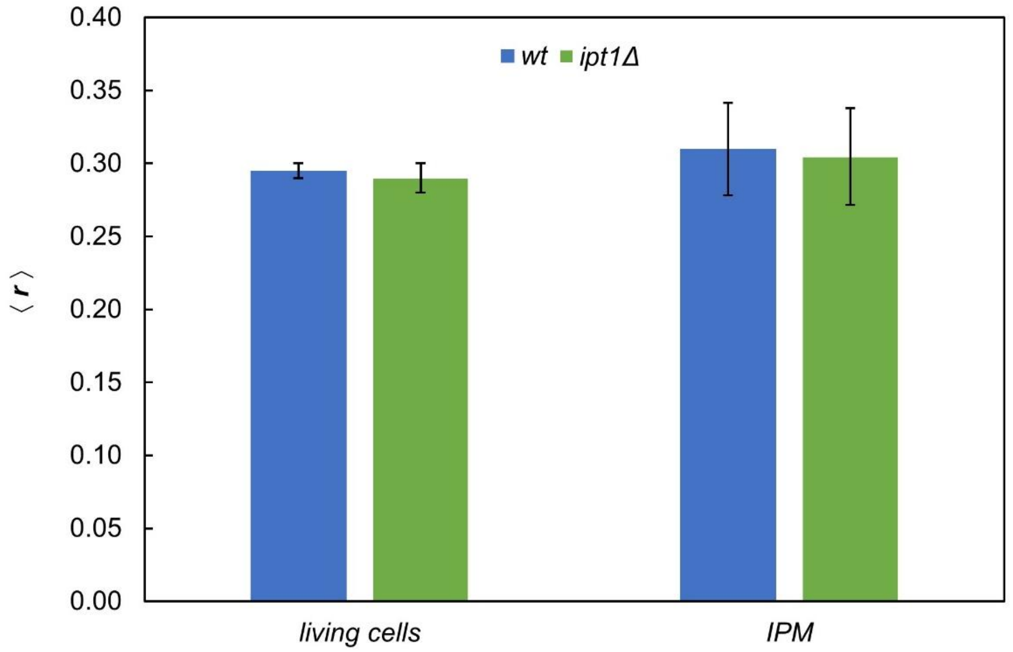

Figure S4: The deletion of *IPT1* gene does not lead to significant differences between intact cells and isolated plasma membrane (IPM) in the sphingolipid-enriched domains. The *t*-PnA steady-state fluorescence anisotropy at 24 °C was obtained as described under "experimental procedures" in suspensions of living *wt* and *ipt1*Δ cells in mid-exponential phase and in IPM. The values are the mean ± S.D. of at least four biological replicates.

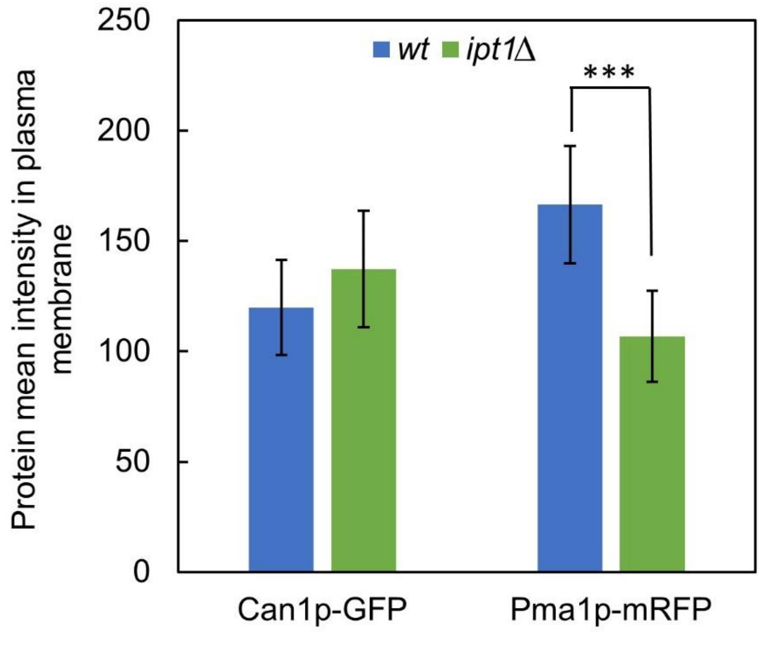

Figure S5: Can1p-GFP and Pma1p-mRFP mean fluorescence intensity in the plasma membrane of *wt* (blue) and *ipt1*Δ (green) cells. The values are the mean ± S.D. of at least four independent biological replicates with a total of at least 200 cells analyzed per replicate. ***$p \leq 0.001$.

Table S1: Parameters describing the fluorescence intensity decay obtained from FLIM experiments of the transformed fluorescent proteins in *S. cerevisiae wt* and *ipt1*Δ plasma membrane. The values are the mean ± S.D. of at least four independent biological replicates with a total of at least 200 cells analyzed per replicate.

Strains	α_1	α_2	τ_1 (ns)	τ_2 (ns)	$\bar{\tau}$ (ns)
***wt* Can1p-GFP**	0.81 ± 0.08	0.19 ± 0.08	1.82 ± 0.09	3.59 ± 0.45	2.10 ± 0.04
***ipt1*Δ Can1p-GFP**	0.83 ± 0.06	0.17 ± 0.06	1.86 ± 0.04	3.60 ± 0.15	2.14 ± 0.06
***wt* Pma1p-mRFP**	0.90 ± 0.01	0.10 ± 0.01	1.57 ± 0.01	4.03 ± 0.16	1.80 ± 0.02
***ipt1*Δ Pma1p-mRFP**	0.95 ± 0.02#	0.05 ± 0.02#	1.64 ± 0.02#	4.45 ± 0.32	1.77 ± 0.03#

# $p<0.001$ *vs wt* Pma1p-mRFP
